# Outcomes of adolescents and young adults (AYA) attending for fertility counselling and oocyte cryopreservation prior to gonadotoxic treatment

**DOI:** 10.1007/s10815-026-03889-z

**Published:** 2026-05-02

**Authors:** Niamh Fee, Niamh Joyce, Maebh Horan, Louise Glover, David Crosby

**Affiliations:** 1 Medicine, University College Dublin, Dublin, Ireland; 2 Merrion Fertility Clinic, Dublin, Ireland; 3 National Maternity Hospital, Dublin, Ireland

**Keywords:** Patient counselling, Fertility preservation, Oocyte, Anti-Müllerian hormone, Antral follicle count

## Abstract

**Purpose:**

Adolescents and young adults (AYA) who receive gonadotoxic treatment are at risk of future infertility. Fertility preservation is recommended to improve long-term quality of life, but outcomes are less well characterised in AYA than in adults. This study sought to analyse oocyte cryopreservation procedures, outcomes and ethical considerations, to improve counselling for AYA patients and their families.

**Methods:**

Single-centre, retrospective observational cohort study of AYA patients (14–25 years) referred for fertility counselling and oocyte vitrification prior to gonadotoxic treatment between July 2018 and July 2025.

**Results:**

Of 47 referrals received, 37 patients initiated ovarian stimulation, and 36 underwent oocyte cryopreservation. Oncological indications included haematological malignancy (43%), sarcoma (30%) and neurological (9%); benign conditions included aplastic anaemia (6%), neurofibromatosis (2%) and desmoid tumour (2%). The median age of patients who underwent ovarian stimulation was 16 years (range 13–24), with median AMH of 15 pmol/L (range 5.1–81.7 pmol/L). Ultrasound follicle tracking was performed transvaginally in 8/37 (21.6%) and transabdominally in 29/37 (78.4%); transvaginal oocyte collection was performed in 34/36 (94.4%) and laparoscopic in 2/36 (5.63%). Median number of oocytes cryopreserved was 11 (range 0–31), with oocyte maturity rate of 80.5% (range 50–100%).

**Conclusions:**

Fertility preservation in post-pubertal AYA patients is feasible, and transabdominal ultrasound monitoring can be used successfully where transvaginal monitoring is not appropriate. Multidisciplinary input is important to ensure fertility preservation can be performed safely, particularly for those with haematological malignancies. To enable clinicians to appropriately counsel young patients, follow-up studies of livebirth outcomes will be important in the long term.

## Introduction

Infertility is predicted to affect one in six people worldwide [1]. Survival rates for adolescents and young adults diagnosed with cancer continue to improve; the 5-year relative survival rate has increased from 58% during the mid-1970s to 85% among children and from 68 to 87% among adolescents [2]. Cancer treatment modalities can negatively impact reproductive potential through inadvertent injury to the hypothalamic-pituitary axis and to reproductive organs themselves [3–5]. Most survivors of cancer treatment express a desire to have children and the potential loss of future fertility can be a source of significant anxiety and stress [6].

International guidelines recommend fertility preservation for treatments with a high risk of gonadotoxicity [7]. There are also benign conditions in the AYA age groups, including haematological and immunological disorders, which may require gonadotoxic treatment, and these patients can also benefit from fertility preservation [8]. In Ireland, FP services (sperm freezing and oocyte cryopreservation) are funded through the state healthcare system for adults 18 years and over with a cancer diagnosis, but an equivalent service has yet to be established for AYA patients. Since 2020, FP for AYA patients has been provided at a single specialist centre affiliated with a tertiary teaching maternity hospital, in collaboration with paediatric and AYA healthcare professionals and funded through a charitable grant [9].

While fertility preservation outcomes for adult women have been well studied, several recent publications have highlighted the need for additional, larger studies of ovarian stimulation and oocyte vitrification in the AYA population [10, 11]. The goal of this study is to describe the multidisciplinary approach to care of AYA patients referred to our fertility centre prior to undergoing gonadotoxic treatment and to report stimulation outcomes in and the safety of oocyte cryopreservation for FP in AYA patients.

## Materials and methods

### Study setting and data collection

This was a single-centre observational cohort study of adolescents and young adults under the age of 25 who were referred for fertility counselling and potential oocyte cryopreservation prior to gonadotoxic treatment between July 2018 and July 2025. This service was funded through the Irish Cancer Society for those up to the age of 18 years and by the Health Service Executive (HSE) for those aged 18 to 25 years. Data was retrieved from a prospectively managed clinical database. Patient data collected included age, anti-Müllerian hormone (AMH), antral follicle count (AFC), age at menarche, medical diagnosis and use of contraception.

### Referral criteria

All patients referred for fertility assessment and possible fertility preservation must be post-menarchal and the aim for the gonadotoxic treatment should be curative. The referring medical team primarily determined that the treatment recommended was at intermediate or high risk of gonadotoxicity using criteria published by the European Society for Medical Oncology [12]. Fertility assessment was discussed with the patient and patients who qualified were offered a referral to a fertility specialist for review. Psychological maturity was assessed by the referring medical team and all those referred were deemed to be appropriately mature to understand and consent to possible oocyte cryopreservation.

### Preprocedural assessment

On initial review, patients underwent testing of ovarian reserve which included both AMH concentration (pmol/L) and antral follicle count by pelvic ultrasound assessment. All patients met with a fertility specialist who had training in oncological fertility preservation and paediatric and adolescent gynaecology. A specialist fertility nurse experienced in oncological fertility preservation also met with the patient and caregivers with support offered virtually following the consultation. Video materials on medication administration were provided to patients and their caregivers following in person instruction to assist with any possible queries regarding administration.

All patients who decided to proceed with oocyte cryopreservation had a consultation with a consultant anaesthesiologist to assess suitability for deep sedation, or suitability for general anaesthetic for those undergoing laparoscopic oocyte retrieval.

### Oocyte cryopreservation cycles

Days of stimulation, cumulative gonadotrophin dose, route of ultrasound monitoring, peak estradiol level (pmol/L) and route of oocyte retrieval were recorded. Total number of oocytes collected, total mature metaphase II (MII) oocytes frozen, dropout rate and complications were collected from electronic patient chart. All cycles were antagonist protocol with recombinant FSH doses determined by ovarian reserve testing. Monitoring was performed with both ultrasound assessment of follicular development and circulating estradiol (pmol/L) levels. Final oocyte maturation triggered with either buserelin acetate alone or in combination with recombinant human chorionic gonadotropin when there was a low risk of ovarian hyperstimulation syndrome. Oocyte retrieval took place 36 h later and was either transvaginally with deep sedation or laparoscopically.

### Statistical analysis

Statistical analysis was performed using GraphPad Prism version 10.0.0 for Windows (GraphPad Software, Boston, MA, USA, www.graphpad.com). Results are presented as mean ± standard deviation or median with range (minimum to maximum), with 95% confidence intervals included where indicated.

### Ethical approval

This was a retrospective audit of services and safety outcomes to improve service provision. Approval was provided by the Clinical Director; ethical approval from a national ethics committee was not required.

## Results

### Patient clinical characteristics

Overall, 47 patients were referred for fertility counselling and possible oocyte preservation between July 2018 and July 2025. Patient demographics and ovarian reserve assessments are presented in Table 1. Of those who attended for fertility assessment, *n* = 37/47 (79%) decided to proceed with oocyte cryopreservation. All of those who did not proceed with oocyte cryopreservation were offered follow-up in an AYA survivor programme. Under this programme, survivors aged 18–26 years are offered fertility assessment and counselling 1 year post completion of treatment, and may consider fertility preservation at that time if appropriate and medically indicated.

**Table 1:** Patient demographics and ovarian reserve assessment

Characteristic	All referred (*n* = 47)	OV cycle started (*n* = 37)	Did not proceed to OV (*n* = 10)
Age (years)			
Referral	16 (12–24)	16 (13–24)	14 (12–17)
Menarche	12 (10–15)	12 (10–15)	11 (10–13)
BMI (kg/m^2^)	23.3 (16.7–33.4)	23.3 (16.7–33.4)	22.5 (17.4–25.1)
Contraception			
No	41 (87.2%)	31 (83.8%)	10 (100%)
Yes	6 (12.8%)	6 (16.2%)	0 (0%)
Type of contraception			
Barrier	1 (2.1%)	1 (2.1%)	0 (0%)
Combined oral contraceptive pill	3 (6.4%)	3 (6.4%)	0 (0%)
Copper coil	1 (2.1%)	1 (2.1%)	0 (0%)
Contraceptive implant	1 (2.1%)	1 (2.1%)	0 (0%)
Barrier	1 (2.1%)	1 (2.1%)	0 (0%)
Ovarian reserve tests			
AMH (pmol/L)	15.1 (0.07–81.7) 95% CI 10.4–20.1	15.1 (5.1–81.7) 95% CI 10.5–20.8	10.4 (0.07–75) 95% CI 0.07–75
Antral follicle count (AFC)	14 (3–41) 95% CI 12–15	14 (3–41) 95% CI 12–17	10 (9–14) 95% CI 9–14
Diagnosis			
Haematological malignancy (AML ALL, lymphoma, myelodysplastic, post-transplant lymphoproliferative disorder)	20 (42.6%)	17 (45.9%)	3 (30%)
Gynaecological malignancy (ovarian small cell ovarian, cervical)	2 (4.3%)	1 (2.7%)	1 (10%)
Benign haematological (aplastic anaemia)	3 (6.4%)	2 (5.4%)	1 (10%)
Sarcoma (mesenchymal chondrosarcoma, Ewing’s, rhabdomyosarcoma, osteosarcoma)	14 (29.8%)	11 (29.7%)	3 (30%)
Neurological malignancy (oligodendroglioma, suprasellar glioma, medulloblastoma)	4 (8.5%)	3 (8.1%)	1 (10%)
Head and neck malignancy (thyroid)	1 (2.1%)	1 (2.7%)	0 (0%)
Skin malignancy (melanoma)	1 (2.1%)	0 (0%)	1 (10%)
Other (neurofibromatosis, desmoid tumour)	2 (4.3%)	2 (5.4%)	0 (0%)

Results are depicted as median and range (95% confidence interval given in parenthesis) or *n* (%). *AMH*, anti-Müllerian hormone

The median age of patients referred was 16 years, with a range of 12–24 (Table 1). All patients were post-menarche with a median age at menarche of 12 (range 10–15 years). The majority of patients were not using contraception (*n* = 41/47; 87.2%). Of those patients who were *n* = 6/47 (12.8%), forms of contraception used included barrier, combined oral contraceptive pill, copper coil or the contraceptive implant. Ovarian reserve testing revealed a median AMH of 15.1 pmol/L (range 0.07–81.7 pmol/L), and a median antral follicle count of 14 (range of 3–41). Two patients had previously undergone chemotherapy for a haematological malignancy but had now relapsed, and were referred for fertility preservation prior to commencing further treatment. One of these patients presented with an AMH of 0.07 pmol/L at time of referral, and oocyte cryopreservation was not recommended.

### Clinical indications for fertility preservation

Indications for referral are shown in Table 1. The majority of AYA patients had been diagnosed with a haematological malignancy *n* = 20/47 (42.6%), followed by sarcoma *n* = 14/47 (29.8%) and neurological malignancy *n* = 4/47 (8.5%). Other malignant diagnoses included gynaecological malignancy *n* = 2/47 (4.3%), thyroid malignancy *n* = 1/47 (2.1%) and melanoma *n* = 1/47 (2.1%). Benign conditions included aplastic anaemia *n* = 3/47 (6.4%), neurofibromatosis *n* = 1/47 (2.1%) and a desmoid tumour *n* = 1/47 (2.1%).

For those patients *n* = 10/47 (21.3%) who did not proceed with oocyte preservation, reasons included urgent need to start treatment *n* = 4/10 (40%), patient preference *n* = 2/10 (20%), low risk of treatment-related gonadotoxicity *n* = 2/10 (20%), previous gonadotoxic treatment with extremely low AMH *n* = 1/10 (10%) and a high-risk urogenital anomaly *n* = 1/10 (10%).

### Ovarian stimulation and oocyte vitrification outcomes

Of the 47 AYA patients referred, 37 (79%) proceeded with controlled ovarian stimulation for oocyte cryopreservation; cycle outcomes are presented in Table 2. Median time from first clinic visit to oocyte retrieval was 14 days. Mean number of days of stimulation was 12.9 (SD ± 2.6) with a mean cumulative gonadotrophin dose of 3436 ± 1632 IU. Mean peak estradiol level was 8760 ± 6474 pmol/L. One patient commenced stimulation but did not progress to oocyte retrieval due to disease progression. Ultrasound monitoring was performed transabdominally in *n* = 29/37 (78.4%) and via transvaginal route in *n* = 8/37 (21.6%), as determined by both age and patient acceptability. The route of oocyte retrieval was transvaginal in *n* = 34/36 (94.4%) and laparoscopic in *n* = 2/36 (5.6%). Laparoscopic oocyte retrieval was required in these patients due to the presence of malignant masses within the vagina due to cervical cancer (*n* = 1) and lymphoma (*n* = 1). The mean number of oocytes collected was 15 ± 8, including a mean of 12 ± 7 mature metaphase II (MII) oocytes. Overall oocyte maturity rate was 80.5% (range 50–100%). One patient underwent oocyte retrieval with no oocytes collected. This patient had a diagnosis of clival chordoma with suboptimal follicular development and subsequent diagnosis of hypopituitarism. The cycle outcomes of the two patients who underwent laparoscopic oocyte collection are shown in Table 2.

**Table 2:** Clinical outcomes from oocyte cryopreservation cycles

Characteristic		Total (*n* = 36)
Days of ovarian stimulation	Mean ± SD	12.9 ± 2.6
	Range	8–21
	95% CI	12.0–13.7
Cumulative gonadotrophin dose (IU)	Mean ± SD	3436 ± 1632
	Median	3200
	95% CI	2884–3989
Peak estradiol (pmol/L)	Mean ± SD	8760 ± 6474
	Range	367–23,269
	95% CI	6570–10,951
Dropout rate	*n* (%)	1 (2.7%)
Number of oocytes retrieved	Mean ± SD	14.8 ± 8.3
	Median (range)	13 (0–37)
	95% CI	12.0–17.6
Metaphase II oocytes retrieved	Mean ± SD	11.9 ± 6.7
	Median (range)	11 (0–31)
	95% CI	9.6–14.2
% metaphase II oocytes	Overall	80.5%
	Range	50–100%
Route of ultrasound monitoring (*n* = 37)	Transvaginal	8 (21.6%)
	Transabdominal	29 (78.4%)
Route of oocyte collection (*n* = 36)	Transvaginal	34 (94.4%)
	Laparoscopic	2 (5.6%)
Complications	Hospital admission	2 (5.4%)
	Platelet transfusion	3 (8.1%)
	OHSS	0 (0%)
Laparoscopic oocyte collection (*n* = 2)		*N* = 2
Patient one	Age	24
	Diagnosis	Cervical Ca
	AMH	9.36
	Days of stimulation	14
	Cumulative FSH dose	4200
	Peak estradiol	10,641
	Number of oocytes	7
	Number of MII frozen	6
	Complications	Nil
Patient two	Age	22
	Diagnosis	Lymphoma
	AMH	11.2
	Days of stimulation	14
	Cumulative FSH dose	4900
	Peak estradiol	17,639
	Number of oocytes	8
	Number of MII frozen	4
	Complications	Nil

Results are given as mean ± standard deviation, *n* (%) and range (minimum to maximum). *IU*, international units; *OHSS*, ovarian hyperstimulation syndrome

**Fig. 1:**
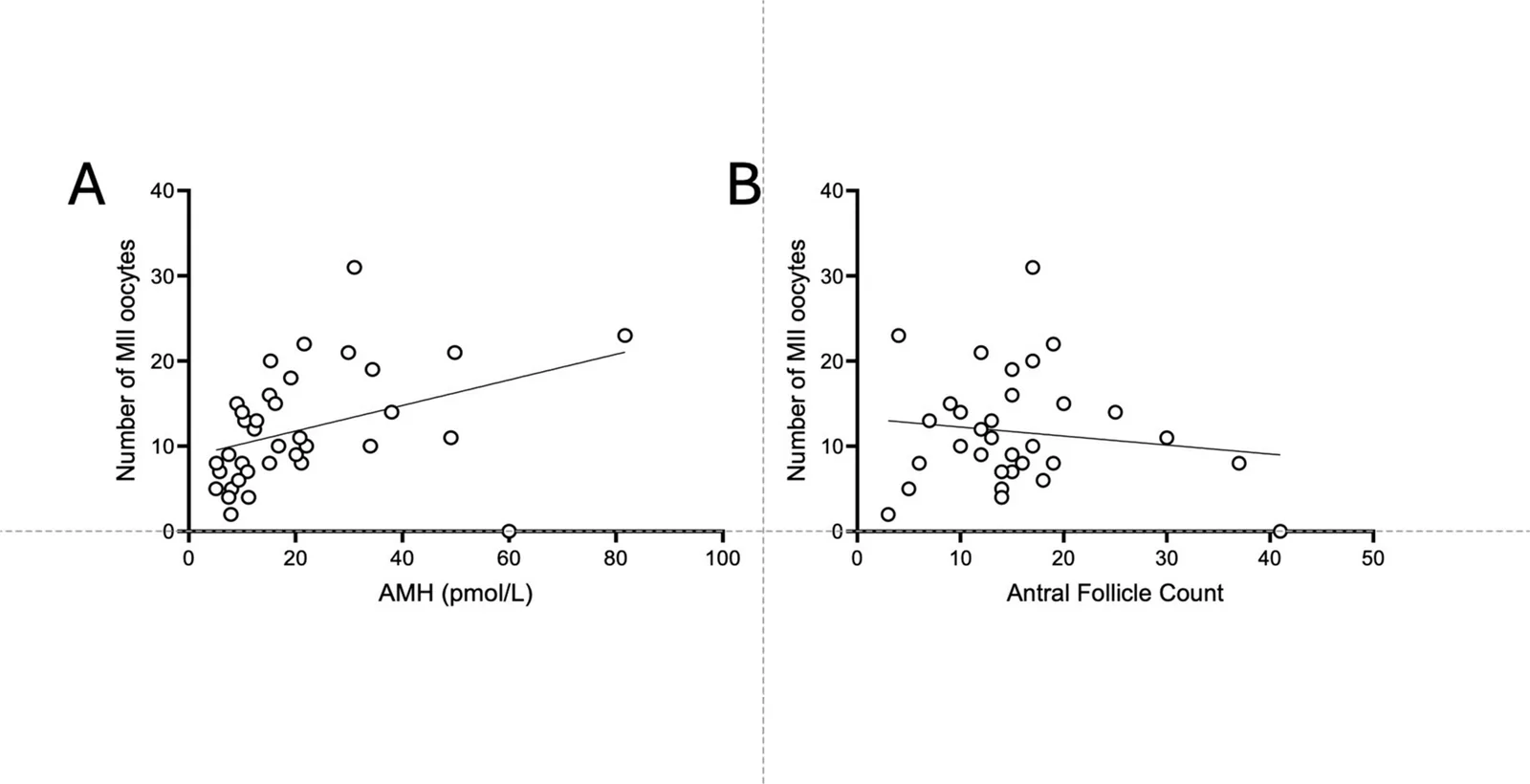
Correlation of ovarian reserve parameters with number of oocytes cryopreserved. Scatter plots showing the relationship between number of MII oocytes and AMH levels (**A**) and antral follicle count (**B**)

**Fig. 2:**
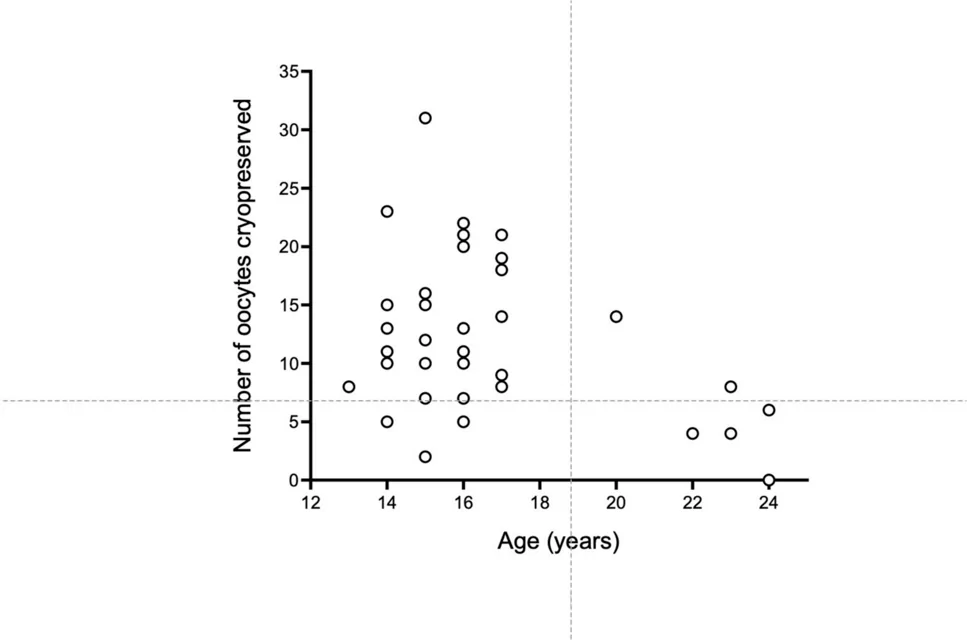
Number of oocytes cryopreserved by age in AYA patients aged 13–24 years

Further analysis was performed to assess the relationship between oocyte yield and baseline measures of ovarian reserve in AYA patients. Spearman correlation analysis revealed that AMH level was positively associated with MII oocyte yield (*r* = 0.5365; 95% CI 0.2430 to 0.7400, *p* = 0.0007), whereas AFC was not significantly associated with MII oocyte yield (*r* = 0.01233, 95% CI − 0.3418 to 0.3634, *p* = 0.9457), as shown in Fig. 1. Notably, AMH and AFC were not found to correlate in this AYA patient cohort (*r* = 0.1749, 95% CI − 0.1678 to 0.4798, *p* = 0.3006). The number of oocytes cryopreserved in patients age 13–24 is shown in Fig. 2. For patients who had oocytes to cryopreserve, mean oocyte number did not significantly differ between those aged less than 18 years (*n* = 30; 13 ± 7 oocytes) and those aged 18 or over (*n* = 5; 7 ± 4 oocytes, *p* = 0.06).

Oocyte cryopreservation outcomes by diagnosis are shown in Table 3. Mean AMH was higher in patients with diagnosis of sarcoma (*n* = 10) and associated with a higher mean number of oocytes cryopreserved (17 ± 9) in this patient group when compared with haematological (*n* = 17 patients, 12 ± 5 oocytes; *p* = 0.07) or other conditions (*n* = 9, 9 ± 5 oocytes; *p* = 0.03). There were fewer frozen oocytes in the benign condition patient group when compared to the patients with a malignant condition (*n* = 32, 12 ± 7 oocytes; *p* = 0.78).

**Table 3:** Oocyte vitrification outcomes categorised by diagnosis (*n* = 36)

Characteristic	Haematological malignancy	Sarcoma	Other (benign haematological, neurological, head/neck, skin, etc.)
Total *n* (%)	17 (47%)	10 (28%)	9 (25%)
Age (years)			
Mean ± SD	16.7 ± 2.5	16.1 ± 3	17.4 ± 3.9
Median (range)	16 (13–23)	15.5 (14–24)	15 (14–24)
AMH (pmol/L)			
Mean ± SD	16 ± 9.3	33.4 ± 23.3	16.4 ± 14.8
Median	15.1 (5.1–38)	25.8 (8–81.7)	10 (7.5–49.1)
Antral follicle count			
Mean ± SD	15 ± 7	16 ± 11	14 ± 9
Median	15 (7–37)	15 (4–41)	13 (3–30)
Oocytes retrieved			
Mean ± SD	13 ± 6	20 ± 11	13 ± 6
Median	11 (7–27)	23 (10–37)	14 (3–20)
Oocytes vitrified			
Mean ± SD	12 ± 5	17 ± 9	9 ± 5
Median	10 (5–24)	19 (0–31)	10 (2–15)

Results are depicted as mean ± standard deviation, median (range), *n* (%); 95% confidence interval given in parenthesis. *AMH*, anti-Müllerian hormone

### Adverse outcomes

No major complications or incidences of ovarian hyperstimulation syndrome were observed in this group. Three patients (8.1%) required a platelet transfusion prior to oocyte retrieval due to thrombocytopenia associated with their haematological malignancy. Two patients (5.4%) were admitted to hospital for observation post oocyte retrieval.

## Discussion

Over the last 20 years, a number of guidelines for management of fertility risks in adolescents and young adults (AYA) have been published [7, 8, 13] that highlight the specialised, interdisciplinary approach needed for this patient cohort. Risks associated with ovarian stimulation for FP in AYA patients include delayed initiation of cancer treatment and the potential effect of stimulation protocols that increase oestrogen levels on hormone-dependent malignancies [14]. Ovarian stimulation and oocyte retrieval require the use of ultrasound scans that are typically performed transvaginally, requiring a level of physical and psychological maturity, though transabdominal scanning is an option for this group. Studies have indicated that oocyte quality and ploidy status may be impacted by young age [15] and there is little long-term follow-up data available on clinical outcomes of oocytes cryopreserved in adolescence [16].

Multidisciplinary input and psychosocial supports are also critical, as hormonal and fertility concerns can affect relationship building and sexuality in a group who may already be struggling with the other effects of their diagnosis and treatment [13].

Fertility preservation and counselling in AYA patients prior to undergoing gonadotoxic treatment is an important aspect of patient care and survivorship planning. The outcomes from our patient group illustrate the feasibility and safety of providing fertility counselling and oocyte preservation to the AYA population. We have shown that ovarian stimulation can successfully be monitored with transabdominal ultrasound along with estradiol levels for patients where transvaginal monitoring is not appropriate. All patients who had transabdominal monitoring of ovarian stimulation did consent to transvaginal oocyte retrieval, and there were no concerns raised by patients or caregivers regarding this and the retrievals were uncomplicated. We did find this cohort of patients that antral follicle count did not correlate with significantly with MII oocyte yield. This finding can potentially be explained by the majority of ultrasound scans being performed transabdominally (*n* = 29, 78.4%), which can result in an underappreciated AFC. Anti-Müllerian hormone was correlated with oocyte yield and is therefore a useful marker in this patient population to aid counselling regarding possible fertility preservation outcomes.

We did note in this young patient group that those undergoing gonadotoxic treatment for a benign condition appeared to yield slightly fewer mature oocytes when compared to those who had a malignant diagnosis. Fertility preservation in patients with benign diseases can prove more challenging, due to their long-term condition or previous medical treatment affecting their ovarian reserve [17].

A recent publication by Brun et al. [18] gives reassuring data, with similar numbers of mature oocytes in patients under 20 years compared with those over 25 years. In this study, a median of nine mature oocytes was retrieved following ovarian stimulation, somewhat lower than our patient cohort median of 13 mature oocytes. Notably, the study by Brun and co-authors included patients who were undergoing fertility preservation due a genetic or idiopathic risk of premature failure, which may explain the reduced oocyte yield compared to our patient population. Our findings are similar to the study by Latif et al. [11], which reported a median of 12 mature oocytes cryopreserved in a young patient group. In contrast to our study, they demonstrated correlation of both AMH and AFC with oocyte yield.

In a 2019 study by Hipp et al. [19], oocyte yield in adolescents aged under 20 years was compared to oocyte yield in adults aged 20–29, 30–34 and older than 35. This large study, comparing 449 ovarian stimulation cycles from adolescents (< 20) to adult groups, showed a reassuringly similar oocyte yield across all age groups, with a mean of 18 oocytes cryopreserved in those under 20 years. While a low risk of complications in this group was noted, 10% of ovarian stimulation cycles were cancelled due to poor response. This contrasts with our cohort where there was one cancellation (1/37; 3%) due not to poor response, but rather to disease progression and an urgent requirement to prioritise oncological treatment.

Our study illustrates the importance of access to multidisciplinary teams. There were three patients who required platelet transfusion immediately prior to oocyte retrieval and two patients who required admission to hospital overnight for monitoring post oocyte retrieval. The availability of subspecialists, including haematologists and anaesthesiologists, along with the availability of a blood bank are needed for this population to provide a safe service. For the small number of patients who require laparoscopic oocyte collection, availability of an operating theatre, anaesthesiologists, nursing staff and laparoscopic expertise with oocyte retrieval is also required. Transvaginal oocyte retrieval is the preferred method for oocyte collection due to better visualisation of follicles with reduced risk of complications related to laparoscopic surgery and general anaesthesia [20]. The two patients in our study who underwent laparoscopic egg collection did retrieve fewer eggs (range 7–8) relative to the mean yield in this overall cohort (15 ± 8 oocytes). Lower yield of mature oocytes following laparoscopic retrieval has been shown in previous studies [21]. However, due to the many potential factors which can reduce predicted oocyte yield at egg collection, this cannot be attributed solely to laparoscopic collection.

There are several limitations to the study. This is a review of AYA oocyte cryopreservation outcomes at a single centre and has not been compared to outcomes from an adult cohort at the same centre. Direct comparison with a general fertility adult population presents several challenges due to notable differences between the AYA and healthy adult fertility groups, including the use of a random start ovarian stimulation protocol in AYA patients and potential impact of disease on hormonal response in young patients [22]. This highlights the need for additional published studies on adolescent COS outcomes to help further delineate these nuances. As ovarian tissue cryopreservation (OTC) is not performed in this country, there is no national OTC data to compare against outcomes of oocyte cryopreservation. Due to the small numbers of those undergoing laparoscopic oocyte collection in our study, no definitive conclusions can be made regarding oocyte yield compared to transvaginal oocyte collection. Similarly, while slight differences were noted in oocyte yields between various conditions, patient numbers within these groups were small and a larger population would be needed to corroborate these observations.

Livebirth rates following oocyte preservation in this age group remain relatively unknown as much of the data available relates to patients over the age of 25 years undergoing social egg freezing [23]. The rate of aneuploidy is higher at both the younger and older ages groups [15], a paradigm that also needs to be discussed during counselling as livebirth rates from oocytes frozen at a young age may be decreased. Looking specifically at patients who preserve oocytes prior to cancer treatment, the livebirth rate (LBR) in one systematic review was 32%. However, within the eight studies included in this review, the age range at time of oocyte cryopreservation was 15–45 years, making it challenging to determine a potential LBR for those younger age groups [24]. The largest study in this group comprised 1073 patients who underwent oocyte freezing prior to oncology treatment, only 7.4% of whom had returned to use their oocytes [25]. Given that the median number of metaphase II oocytes cryopreserved by our patient cohort was 12, this gives a reasonable prospect for a resulting live birth. However, the need exists for a long-term survivorship register in order to further assess pregnancy outcomes such as aneuploidy rate, miscarriage, maternal and neonatal outcomes. This ongoing collection of data would be highly beneficial for both patients and their caregivers, as it would help inform decision-making regarding fertility preservation and would also aid in preconception counselling when a patient returns to discuss use of their cryopreserved oocytes.

For those patients who are unable to avail of oocyte cryopreservation, ESHRE recommends ovarian tissue cryopreservation (OTC) [8]. Reasons to avail of OTC rather than oocyte cryopreservation can include young age and lack of sexual maturity (prepubertal), the urgent need to commence treatment as a priority over the time delay required for ovarian stimulation, or patient preference over oocyte cryopreservation. The American Society for Reproductive Medicine no longer defines OTC as experimental [26] due to continued data on its safety and associated fertility outcomes. The spontaneous livebirth rate from subsequent ovarian tissue transplantation (OTT) from OTC has been reported at 33% with a livebirth following IVF in OTT as 19% [16, 24]. OTC requires both surgical expertise and specific laboratory training and equipment distinct from a standard IVF laboratory, and this is currently unavailable in Ireland.

Despite international consensus on the provision of fertility preservation in AYA prior to gonadotoxic treatment, inconsistencies remain in the provision of services in Europe [8, 27, 28]. Our service is currently funded largely by a charitable organisation, and there is a pressing need to move to public sector funding to ensure its continued development. There is no variation in the care of who referred to this service under the age of 18 years as all treatment is performed at a single centre, which can be a challenge in other countries [27]. However, as we are unable to perform OTC at this time, we cannot offer the full spectrum of fertility preservation to those undergoing gonadotoxic treatment.

For adolescents, young adults and their parents or caregivers who are facing decisions around fertility preservation prior to gonadotoxic treatment, access to appropriately trained nursing and clinical healthcare providers is essential. Studies examining these patient groups and their decision-making regarding fertility and gonadotoxic treatment have shown that factors associated with low regret are high-quality, timely consultations with a fertility specialist [29]. By performing appropriate, pre-procedure counselling and having specialist nursing staff available for these patients and their families, we can ensure appropriate support for this complex group.

## Conclusion

Fertility counselling and preservation for AYA patients prior to receiving gonadotoxic treatment is a safe and effective fertility preservation option. Ovarian stimulation and oocyte retrieval requires a multidisciplinary team approach, and the low complication rate and oocyte yield in this patient group is reassuring for clinicians. Long-term follow-up studies of livebirth outcomes will be important to facilitate appropriate counselling of patients and their families.

## Data Availability

Data regarding any of the subjects in the study has not been previously published unless specified. Data will be made available to the editors of the journal for review or query upon request.
